# Assessment of Physician Sleep and Wellness, Burnout, and Clinically Significant Medical Errors

**DOI:** 10.1001/jamanetworkopen.2020.28111

**Published:** 2020-12-07

**Authors:** Mickey T. Trockel, Nikitha K. Menon, Susannah G. Rowe, Miriam T. Stewart, Randall Smith, Ming Lu, Peter K. Kim, Mariah A. Quinn, Elizabeth Lawrence, Daniel Marchalik, Heather Farley, Patricia Normand, Mila Felder, Jessica C. Dudley, Tait D. Shanafelt

**Affiliations:** 1 Stanford University School of Medicine, Palo Alto, California; 2 Boston Medical Center, Boston, Massachusetts; 3 Children’s Hospital of Philadelphia, Philadelphia, Pennsylvania; 4 Physician Affiliate Group of New York, New York, New York; 5 University of Wisconsin School of Medicine, Madison; 6 University of New Mexico School of Medicine, Albuquerque; 7 MedStar Health, Columbia, Maryland; 8 Christiana Care Health System, Middletown, Delaware; 9 Rush University Medical Center, Chicago, Illinois; 10 Advocate Christ Medical Center, Oak Lawn, Illinois; 11 Brigham and Women's Hospital–Partners HealthCare, Boston, Massachusetts

## Abstract

**Importance:**

Sleep-related impairment in physicians is an occupational hazard associated with long and sometimes unpredictable work hours and may contribute to burnout and self-reported clinically significant medical error.

**Objective:**

To assess the associations between sleep-related impairment and occupational wellness indicators in physicians practicing at academic-affiliated medical centers and the association of sleep-related impairment with self-reported clinically significant medical errors, before and after adjusting for burnout.

**Design, Setting, and Participants:**

This cross-sectional study used physician wellness survey data collected from 11 academic-affiliated medical centers between November 2016 and October 2018. Analysis was completed in January 2020. A total of 19 384 attending physicians and 7257 house staff physicians at participating institutions were invited to complete a wellness survey. The sample of responders was used for this study.

**Exposures:**

Sleep-related impairment.

**Main Outcomes and Measures:**

Association between sleep-related impairment and occupational wellness indicators (ie, work exhaustion, interpersonal disengagement, overall burnout, and professional fulfillment) was hypothesized before data collection. Assessment of the associations of sleep-related impairment and burnout with self-reported clinically significant medical errors (ie, error within the last year resulting in patient harm) was planned after data collection.

**Results:**

Of all physicians invited to participate in the survey, 7700 of 19 384 attending physicians (40%) and 3695 of 7257 house staff physicians (51%) completed sleep-related impairment items, including 5279 women (46%), 5187 men (46%), and 929 (8%) who self-identified as other gender or elected not to answer. Because of institutional variation in survey domain inclusion, self-reported medical error responses from 7538 physicians were available for analyses. Spearman correlations of sleep-related impairment with interpersonal disengagement (*r* = 0.51; *P* < .001), work exhaustion (*r* = 0.58; *P* < .001), and overall burnout (*r* = 0.59; *P* < .001) were large. Sleep-related impairment correlation with professional fulfillment (*r* = −0.40; *P* < .001) was moderate. In a multivariate model adjusted for gender, training status, medical specialty, and burnout level, compared with low sleep-related impairment levels, moderate, high, and very high levels were associated with increased odds of self-reported clinically significant medical error, by 53% (odds ratio, 1.53; 95% CI, 1.12-2.09), 96% (odds ratio, 1.96; 95% CI, 1.46-2.63), and 97% (odds ratio, 1.97; 95% CI, 1.45-2.69), respectively.

**Conclusions and Relevance:**

In this study, sleep-related impairment was associated with increased burnout, decreased professional fulfillment, and increased self-reported clinically significant medical error. Interventions to mitigate sleep-related impairment in physicians are warranted.

## Key Points

**Question:**

Is sleep-related impairment associated with burnout, professional fulfillment, and self-reported clinically significant medical error in physicians?

**Findings:**

In this cross-sectional study of 11 395 physicians, sleep-related impairment had statistically significant correlations with burnout and professional fulfillment. In a model adjusting for gender, training status, practice specialty, and burnout, moderate, high, and very high sleep-related impairment were associated with 53%, 96%, and 97% greater odds of self-reported clinically significant medical error, respectively, compared with low sleep-related impairment.

**Meaning:**

These findings suggest that interventions to mitigate sleep-related impairment in physicians are warranted.

## Teaser

This survey study examines sleep-related impairment and occupational wellness indicators in physicians to assess the association with self-reported clinically significant medical error, before and after adjusting for burnout.

## Introduction

In a profession in which excessive, often unpredictable work hours are expectations,^1,2,3^ sleep-related impairment is an occupational hazard for physicians.^4,5^ The evidence that inadequate sleep has significant health and cognitive performance consequences is robust.^6,7,8,9^ Sleep deprivation disrupts connectivity and processing within and between the amygdala, anterior cingulate, and medial prefrontal cortex, resulting in emotional dysregulation.^10,11,12,13,14^ Furthermore, sleep deprivation decreases ability to discern^13,15,16^ and mirror emotions,^9^ which may decrease physicians’ capacity for empathy and interpersonal engagement. This could explain previously observed impaired social affection among physician trainees who are sleep deprived.^17^ Insufficient sleep also results in reduced capacity to maintain attention—including dose-dependent attention gaps proportional to increasing hours awake^18,19^—associated with reduced intraparietal sulcus and dorsolateral prefrontal cortex activity.^20,21^ Attention gaps associated with sleep-related impairment^18,19^ may affect physicians’ ability to perform critical cognitive tasks of patient care, including assessment and treatment planning.

A large, increasing body of evidence illustrates the decrements in clinical performance associated with sleep-related impairment. Emergency physicians working night shifts take longer to intubate,^22^ display an increased propensity for error as the shift progresses,^23^ and exhibit a significant decline in cognitive performance after working 5 consecutive night shifts.^24^ Sleep-impaired physicians in training exhibit deficiencies in functional cognition,^25,26^ concentration,^27^ working^27^ and visual memory,^28^ operative dexterity,^29^ vigilance,^30,31^ and ability to discern arrhythmias on an electrocardiogram.^17^ One night of sleep deprivation in physician trainees led to reduction in scores on the examination of the American Board of Family Practice equal to the average difference in scores between first-year and third-year residents.^32^ Sleep-related impairment also results in impaired decision-making,^33,34^ including reduced capacity for risk-benefit analysis^35^ and increased risk-taking behavior.^35,36^ For instance, sleep-deprived residents responding to a case vignette select more risky treatment options.^37^ Both sleep-related impairment and burnout among physicians are associated with increased unsolicited patient complaints,^38^ a factor associated with adverse clinical outcomes and liability risk.^39,40,41^

Although the link between sleep-related impairment and burnout among physicians has been postulated, few studies have evaluated it.^42,43^ A previous report demonstrated an association of sleep-related impairment with burnout in a small sample of predominantly physicians in postgraduate training.^43^ To our knowledge, no previous study has assessed the association of sleep-related impairment with self-reported clinically significant medical errors (ie, errors resulting in patient harm). The objectives of this study were to assess the association between sleep-related impairment and occupational wellness indicators—work exhaustion, interpersonal disengagement, overall burnout, and professional fulfillment—in a large sample of physicians in postgraduate training and attending physicians at academic-affiliated medical centers and to assess the associations of sleep-related impairment and burnout with self-reported clinically significant medical error. This study also explored differences in sleep-related impairment by medical specialty and between attending physicians and postgraduate medical trainees in the same specialty.

## Methods

### Context and Sample

The Physician Wellness Academic Consortium is an expanding group of academic-affiliated institutions in the United States, committed to longitudinal multicenter assessment of clinician well-being. The assessment supports the larger Physician Wellness Academic Consortium mission to use standardized longitudinal survey data to inform iterative program development and evaluation to improve clinician wellness at the organizational and consortiumwide levels. Throughout the year, the consortium convenes monthly, with 10 teleconference sessions and 2 in-person conferences to discuss survey findings, best practices, and intervention strategies. The data for this analysis were obtained from 11 institutions surveyed between November 2016 and October 2018. These institutions are predominantly in the northeast United States, with some representation in the West and Midwest. Data were deidentified by the third-party survey administrator before delivery to 1 of us (M.T.T.) for analysis. The institutional review board at Stanford University determined this project using completely deidentified data to be exempt from further review. Partnering institutions consented to secondary analysis of the aggregated data set. This article follows the Strengthening the Reporting of Observational Studies in Epidemiology (STROBE) reporting guideline for reporting cross-sectional studies.

### Measures

Sleep-related impairment was assessed with the well-validated and widely used Patient-Reported Outcomes Measurement Information System (PROMIS) 8-item scale that assesses tiredness, alertness, sleepiness, and functional deficits related to inadequate sleep. Summed 5-point Likert scale item scores total to a range of 8 to 40. We used published sleep-related impairment T scores to create population-based quartile cut points (raw score of 12 indicates a T score of 43.6 and the 26th percentile; raw score of 16 indicates a T score of 50.3 and the 51st percentile; raw score of 22 indicates a T score of 61.5 and the 76th percentile). These published raw score to T score conversions are based on national benchmark data from a combination of general and clinical populations.^44,45,46^ Using these quartile cut points from published benchmarks, we categorized scores from 8 to 11 as negligible levels of sleep-related impairment, 12 to 15 as low, 16 to 21 as high, and 22 to 40 as very high.

The Physician Wellness Academic Consortium assesses burnout with the Professional Fulfillment Index,^43^ which measures burnout in the following 2 domains: a 6-item scale that measures interpersonal disengagement and a 4-item scale that measures work exhaustion. The 2 burnout subscales are combined to derive an overall burnout score. The index also measures professional fulfillment with a 6-item scale. All index items are scored on a 5-point Likert scale. Physician Wellness Academic Consortium benchmark statistics are reported with Professional Fulfillment Index scale scores that are standardized to a range of 0 to 10 to facilitate benchmark comparison for the consortium. Previously published Professional Fulfillment Index scale cut points,^43^ which used a scale from 0 to 4, were converted to cut points on a scale from 0 to 10 by multiplying the previous versions by 2.5. A score of 7.5 or greater on the professional fulfillment scale indicates high professional fulfillment. A score of 3.325 or greater on the subscales for work exhaustion, interpersonal disengagement, or overall burnout indicates high to very high levels of each.

The self-reported medical error scale^43^ assesses the occurrence of self-reported medical errors on a 6-point scale from never to within the last week. For this study, we specified a binary self-reported medical error variable. Specifically, we defined physicians with a clincally significant self-reported medical error as those who indicated “I made a medical error that did result in patient harm” with a response option of within the last year or more recently. Some, but not all, institutions included self-reported medical error questions with their wellness survey.

### Statistical Analysis

Using SPSS statistical software version 25.0 (IBM Corp) for all analyses, we provided descriptive data, including mean (SD) and median (interquartile range) for sleep-related impairment, burnout, and professional fulfillment by gender and trainee status for the sample overall and by gender, trainee status, and medical specialty categories. We assessed associations of sleep-related impairment with depersonalization, work exhaustion, burnout, and professional fulfillment by calculating Spearman rank order correlation coefficients for the sample overall and by gender, trainee status, and medical specialty categories. We specified a logistic regression model adjusting for gender, trainee vs attending physician status, medical practice specialty, and burnout level to assess the association of low, high, or very high sleep-related impairment—compared with negligible sleep-related impairment—with self-reported clinically significant medical error. Analysis was completed in January 2020. Statistical significance was set at *P* < .05, and all tests were 2-tailed.

## Results

Of 11 institutions, 2 (18%) invited only attending physicians to participate, 1 (9%) invited only postgraduate physicians in training, and 8 (73%) invited both groups. Response rates varied by institution and by training status, with that of attending physicians ranging from 20% to 60% and that of trainees from 38% to 74% across institutions. Overall, of 19 384 attending physicians and 7257 house staff physicians invited to participate, 7700 (40%) and 3695 (51%), respectively, completed sleep-related impairment questions, rendering data from 11 395 physicians available for analyses. Of these, 5279 (46%) self-identified as women, 5187 (46%) as men, and 929 (8%) as other gender or elected not to answer. Because of institution-level variation in inclusion of this domain (8 institutions included), there were self-reported medical error responses from 7762 physicians, of whom 7538 (97%) also completed sleep-related impairment and burnout assessments.

### Sleep-Related Impairment by Medical Specialty in Attending and House Staff Physicians

**Figure 1.**
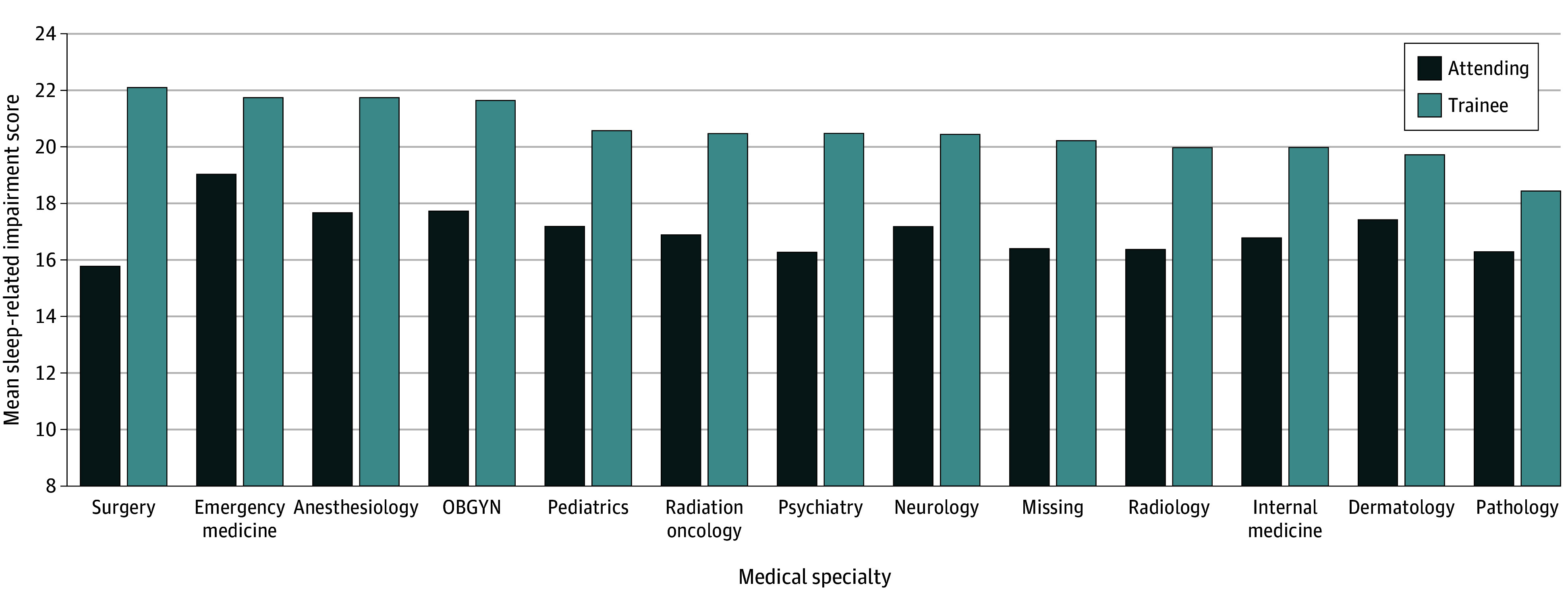
Mean Sleep-Related Impairment Level Among Trainees Compared With Attending Physicians, by Medical Practice Specialty OBGYN indicates obstetrics and gynecology.

The mean (SD) sleep-related impairment scale score in physicians was 16.9 (6.4); among trainee physicians, it was 20.7 (7.4). Sleep-related impairment varied by specialty, gender and training status. Figure 1 demonstrates mean sleep-related impairment levels by attending physician vs trainee status within each of 12 medical specialty categories. Subspecialties are grouped in larger medical specialty categories (eg, ear/nose/throat and ophthalmology are included in the surgery specialty category; obstetrics/gynecology is separated as its own category). Table 1 provides descriptive data for sleep-related impairment, burnout, and professional fulfillment, including number of survey completers and mean (SD) and median (interquartile range) by gender, trainee status, and medical practice specialty. Table 1 also reports the proportion of physicians at or above the PROMIS database upper-quartile cut point score of 22 and the proportions meeting published criteria for high burnout and high professional fulfillment. The data suggest gender differences, which are beyond the scope of this article. Among attending physicians, emergency medicine specialists had the highest sleep-related impairment scores and surgical specialists, the lowest. Attending surgical specialists had mean sleep-related impairment scores below the national normed PROMIS data bank median of 16. In contrast, trainees in surgical specialty training programs had the highest sleep-related impairment of the 12 specialty categories of postgraduate training specialties. Accordingly, surgical specialties have a large observed gap (difference, >0.8 SD units; *P* < .001) in sleep-related impairment between attending and trainee physicians.

**Table 1.** Sleep-Related Impairment, Overall Burnout, and Professional Fulfillment by Gender, Training Status, and Medical Specialty

Participant population	Sleep-related impairment; range, 8-40				Burnout; range, 0-10				Professional fulfillment; range, 0-10			
	No.	Mean (SD)	Median (IQR)	Score, ≥22, No. (%)	No.	Mean (SD)	Median (IQR)	Score, ≥3.3, No. (%)	No.	Mean (SD)	Median (IQR)	Score ≥7.5, No. (%)
**All physicians**												
All genders	11 395	18.09 (6.98)	17.00 (12.00-23.00)	3317 (29.1)	11 166	2.99 (1.99)	2.75 (1.50-4.25)	4337 (38.8)	12 569	6.48 (2.11)	6.67 (5.00-7.91)	4894 (38.9)
Women	5279	18.4 (6.99)	17.00 (13.00-23.00)	1608 (30.5)	4989	3.17 (1.98)	3.00 (1.75-4.25)	2129 (42.7)	5672	6.21 (2.04)	6.25 (5.00-7.50)	1860 (32.8)
Men	5187	17.68 (6.90)	16.00 (12.00-22.00)	1408 (27.1)	5003	2.81 (1.98)	2.50 (1.25-4.00)	1756 (35.1)	5582	6.74 (2.13)	7.08 (5.42-8.33)	2509 (44.9)
Other or missing	929	18.52 (7.24)	17.00 (13.00-24.00)	301 (32.4)	1174	2.98 (2.03)	2.75 (1.25-4.25)	452 (38.5)	1315	6.49 (2.18)	6.67 (5.00-7.92)	525 (39.9)
**House staff physicians, ie, residents and fellows**												
Anesthesiology	245	21.74 (7.38)	21.00 (16.00-26.00)	117 (47.8)	250	3.69 (1.97)	3.50 (2.50-5.00)	127 (50.8)	251	5.76 (2.02)	5.83 (4.58-7.08)	58 (23.1)
Dermatology	53	19.72 (7.80)	17.00 (13.00-25.50)	20 (37.7)	53	3.38 (2.42)	2.75 (1.50-4.62)	21 (39.6)	55	6.36 (2.18)	6.67 (5.00-7.50)	20 (36.4)
Emergency	230	21.74 (7.61)	21.00 (14.00-25.00)	111 (48.3)	233	3.54 (1.98)	3.50 (2.25-4.75)	118 (50.6)	235	6.19 (2.00)	6.25 (4.58-7.50)	78 (33.2)
Medicine	1257	19.98 (7.27)	19.00 (14.00-25.00)	483 (38.4)	1299	3.29 (1.95)	3.25 (2.00-4.50)	586 (45.1)	1303	6.18 (2.08)	6.25 (5.00-7.50)	430 (33.0)
Neurology	126	20.44 (7.60)	19.50 (15.00-26.00)	53 (42.1)	129	3.41 (1.99)	3.25 (2.00-5.00)	55 (42.6)	129	6.25 (2.16)	6.67 (5.00-7.50)	43 (33.3)
OBGYN	155	21.64 (7.08)	22.00 (16.00-26.00)	79 (51.0)	155	3.62 (2.18)	3.50 (2.25-4.75)	81 (52.3)	155	6.23 (2.18)	6.67 (5.00-7.50)	57 (36.8)
Pathology	130	18.44 (6.89)	17.00 (13.00-23.25)	42 (32.3)	131	2.40 (1.95)	2.00 (0.75-3.50)	36 (27.5)	142	6.68 (2.10)	7.08 (5.00-8.02)	61 (43.0)
Pediatrics	350	20.57 (7.09)	20.00 (15.00-25.00)	140 (40.0)	358	3.18 (1.92)	3.00 (1.75-4.50)	158 (44.1)	368	6.28 (2.03)	6.67 (5.00-7.50)	120 (32.6)
Psychiatry	208	20.48 (7.25)	19.00 (15.00-26.00)	81 (38.9)	214	3.72 (2.26)	3.50 (2.25-4.81)	115 (53.7)	213	6.02 (2.32)	6.25 (4.58-7.50)	71 (33.3)
Radiation oncology	30	20.47 (8.90)	18.50 (13.75-26.50)	11 (36.7)	30	3.19 (2.27)	2.75 (1.25-4.75)	11 (36.7)	31	6.30 (2.27)	7.08 (4.58-7.92)	11 (35.5)
Radiology	223	19.97 (7.66)	18.00 (14.00-26.00)	83 (37.2)	225	2.85 (2.13)	2.50 (1.25-3.75)	71 (31.6)	231	6.43 (2.27)	6.67 (5.00-7.92)	87 (37.7)
Surgery	561	22.10 (7.42)	22.00 (16.00-27.00)	286 (51.0)	586	3.55 (2.13)	3.25 (2.00-4.75)	288 (49.1)	591	6.22 (2.19)	6.67 (5.00-7.50)	198 (33.5)
Missing	127	20.22 (7.52)	20.00 (14.00-25,00)	50 (39.4)	139	3.29 (2.04)	3.25 (1.75-4.75)	62 (44.6)	142	5.66 (2.26)	5.83 (4.17-7.50)	41 (28.9)
All house staff	3695	20.65 (7.40)	20.00 (15.00-26.00)	1556 (42.1)	3802	3.35 (2.05)	3.25 (2.00-4.75)	1729 (45.5)	3846	6.18 (2.13)	6.25 (5.00-7.50)	1275 (33.2)
**All medical staff, ie, not trainees**												
Anesthesiology	468	17.71 (6.42)	16.00 (13.00-22.00)	120 (25.6)	389	3.17 (1.96)	3.00 (1.75-4.25)	163 (41.9)	516	6.26 (2.28)	6.67 (5.00-7.92)	189 (36.6)
Dermatology	100	17.39 (6.48)	16.50 (12.00-21.00)	24 (24.0)	75	2.63 (1.79)	2.50 (1.25-4.00)	26 (34.7)	101	7.06 (1.89)	7.50 (6.25-8.33)	53 (52.5)
Emergency	381	19.03 (6.88)	18.00 (14.00-24.00)	132 (34.6)	384	3.16 (1.95)	3.00 (1.75-4.25)	151 (39.3)	436	6.45 (2.00)	6.67 (5.00-7.50)	164 (37.6)
Medicine	2530	16.79 (6.50)	15.00 (11.00-20.00)	581 (23.0)	2494	2.93 (2.00)	2.75 (1.25-4.00)	952 (38.2)	2793	6.50 (2.10)	6.67 (5.00-7.92)	1,116 (40.0)
Neurology	254	17.21 (6.93)	16.00 (12.00-21.25)	63 (24.8)	213	2.77 (2.00)	2.75 (1.25-3.75)	67 (31.5)	266	6.65 (2.06)	7.08 (4.42-7.92)	110 (41.4)
OBGYN	354	17.73 (6.64)	17.00 (12.00-22.00)	96 (27.1)	361	2.91 (1.89)	2.75 (1.50-4.25)	137 (38.0)	417	6.69 (2.08)	7.08 (4.42-7.92)	179 (42.9)
Pathology	174	16.29 (6.91)	15.00 (11.00-20.00)	35 (20.1)	137	2.06 (1.76)	1.50 (0.75-3.25)	33 (24.1)	185	7.15 (1.92)	7.50 (6.25-8.33)	95 (51.4)
Pediatrics	1026	17.15 (6.11)	16.00 (12.00-21.00)	252 (24.6)	934	2.63 (1.80)	2.50 (1.25-3.75)	296 (31.7)	1132	6.68 (1.96)	7.08 (5.42-7.92)	473 (41.8)
Psychiatry	266	16.28 (5.71)	15.00 (12.00-20.00)	49 (18.4)	246	2.73 (2.10)	2.50 (1.00-3.25)	89 (36.2)	340	6.58 (2.15)	6.67 (5.00-7.92)	141 (41.5)
Radiation oncology	87	16.91 (6.61)	15.00 (12.00-21.00)	21 (24.1)	72	2.33 (1.76)	2.00 (1.00-3.50)	19 (26.4)	90	7.38 (1.69)	7.50 (6.25-8.75)	49 (54.4)
Radiology	413	16.41 (6.27)	15.00 (11.00-20.50)	88 (21.3)	386	2.63 (1.78)	2.50 (1.19-3.75)	117 (30.3)	452	6.54 (2.03)	6.67 (5.42-7.92)	169 (37.4)
Surgery	1081	15.81 (6.02)	14.00 (11.00-19.00)	183 (16.9)	979	2.59 (1.92)	2.50 (1.00-3.75)	318 (32.5)	1180	7.05 (2.04)	7.50 (5.83-8.75)	596 (50.5)
Missing	566	16.38 (6.29)	15.00 (11.00-20.00)	117 (20.7)	694	2.76 (1.86)	2.50 (1.25-4.00)	240 (34.6)	815	6.26 (2.17)	6.67 (5.00-7.50)	285 (35.0)
All medical staff	7700	16.86 (6.41)	16.00 (12.00-21.00)	1761 (22.9)	7364	2.80 (1.94)	2.50 (1.25-4.00)	2608 (35.4)	8723	6.61 (2.09)	7.08 (5.41-7.91)	3619 (41.5)

Abbreviations: IQR, interquartile range; OBGYN, obstetrics and gynecology.

### Associations Between Sleep-Related Impairment and Occupational Wellness Indicators

The association between increasing levels of sleep-related impairment and interpersonal disengagement, work exhaustion, burnout, and professional fulfillment is shown in Table 2. Spearman correlations for the overall sample of all physicians were high for associations of sleep-related impairment with burnout components (interpersonal disengagement, *r* = 0.51; *P* < .001; work exhaustion, *r* = 0.58; *P* < .001) and overall burnout (*r* = 0.59; *P* < .001) and moderate with professional fulfillment (*r* = −0.40; *P* < .001). The correlations of sleep-related impairment with interpersonal disengagement across gender and specialty categories of attending physicians and trainees ranged from 0.38 (*P* = .001) among radiation oncology house staff to 0.60 (*P* < .001) among attending pathologists and pediatricians. The correlations of sleep-related impairment with professional fulfillment across gender and specialty categories of attending physicians and trainees ranged from −0.23 (*P* = .03) among radiation oncology house staff to −0.56 (*P* < .001) among attending radiation oncologists.

**Table 2.** Spearman Correlations of Sleep-Related Impairment With Interpersonal Disengagement, Work Exhaustion, Overall Burnout, and Professional Fulfillment by Gender, Training Status, and Medical Specialty^a^

Participant population	Interpersonal disengagement	Work exhaustion	Overall burnout	Professional fulfillment
**All physicians**				
All genders	0.51	0.58	0.59	−0.40
Women	0.48	0.55	0.56	−0.36
Men	0.54	0.61	0.62	−0.44
Other or missing	0.53	0.58	0.59	−0.43
**House staff physicians, ie, residents and fellows**				
Anesthesiology	0.52	0.58	0.59	−0.36
Dermatology	0.58	0.58	0.60	−0.43
Emergency	0.47	0.65	0.58	−0.45
Medicine	0.54	0.63	0.62	−0.46
Neurology	0.57	0.65	0.66	−0.54
OBGYN	0.52	0.60	0.58	−0.43
Pathology	0.60	0.69	0.69	−0.54
Pediatrics	0.60	0.67	0.68	−0.50
Psychiatry	0.55	0.64	0.62	−0.44
Radiation oncology	0.52	0.63	0.59	−0.56
Radiology	0.50	0.65	0.62	−0.49
Surgery	0.56	0.67	0.65	−0.50
Missing	0.50	0.60	0.60	−0.37
All house staff	0.55	0.64	0.63	−0.47
**All medical staff, ie, not trainees**				
Anesthesiology	0.45	0.53	0.53	−0.36
Dermatology	0.48	0.64	0.59	−0.41
Emergency	0.44	0.50	0.49	−0.26
Medicine	0.47	0.57	0.55	−0.36
Neurology	0.52	0.56	0.61	−0.29
OBGYN	0.43	0.55	0.53	−0.40
Pathology	0.48	0.59	0.55	−0.46
Pediatrics	0.40	0.54	0.52	−0.33
Psychiatry	0.54	0.51	0.57	−0.39
Radiation oncology	0.38^b^	0.58	0.52	−0.23^c^
Radiology	0.48	0.52	0.55	−0.34
Surgery	0.54	0.58	0.61	−0.38
Missing	0.44	0.56	0.55	−0.33
All medical staff	0.47	0.56	0.55	−0.36

Abbreviation: OBGYN, obstetrics and gynecology.

^a^ *P* < .001 unless otherwise indicated.

^b^ *P* = .03.

^c^ *P* = .001.

### Associations of Sleep-Related Impairment and Burnout With Self-reported Clinically Significant Medical Error

In a multivariate logistic regression model of data from the combined sample of 7538 attending and trainee physicians who provided data on self-reported medical errors, sleep-related impairment had a dose-related association with the odds of self-reported clinically significant medical error after adjusting for gender, trainee status (vs attending physician), and medical practice specialty. In this adjusted model, compared with low sleep-related impairment, moderate, high, and very high levels of sleep-related impairment were associated with increased odds of self-reported clinically significant medical error, by 66% (odds ratio [OR], 1.66; 95% CI, 1.22 to 2.23), 141% (OR, 2.41; 95% CI, 1.81 to 3.22), and 194% (OR, 2.94; 95% CI, 2.22 to 3.90), respectively. The association of sleep-related impairment with odds of self-reported clinically significant medical error remained significant after controlling for quartile level of burnout. In the model adjusted for gender, trainee status, medical specialty, and burnout level, compared with low sleep-related impairment, moderate, high, and very high levels of sleep-related impairment were associated with increased odds of self-reported clinically significant medical error by 53% (OR, 1.53; 95% CI, 1.12 to 2.09), 96% (OR, 1.96; 95% CI, 1.46 to 2.63), and 97% (OR, 1.97; 95% CI, 1.45 to 2.69), respectively. Each 1-point increase in burnout (scale, 0-10) was associated with a 14% increase in odds of self-reported clinically significant medical error (OR, 1.14; 95% CI, 1.10 to 1.19). Physician trainees had 118% greater odds of self-reported clinically significant medical error compared with attending physicians (OR, 2.18; 95% CI, 1.86 to 2.55).

**Figure 2.**
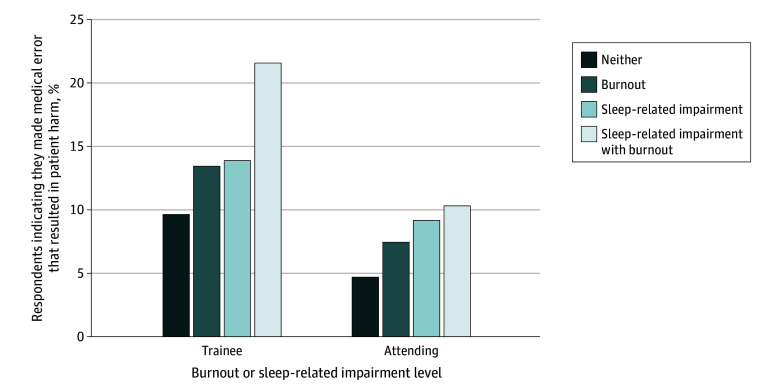
Probability of Error That Resulted in Patient Harm, by Sleep-Related Impairment and Burnout Categories Among 7624 Physicians and Trainee Physicians Sleep-related impairment was defined as a score of 16 or greater on the Sleep-Related Impairment Scale.

Physicians with less sleep-related impairment were less likely to make self-reported clinically significant medical errors. The proportion of all physicians who made a clinically significant mistake was 7.5% of attending physicians (308 of 4087) and 16.0% of trainee physicians (553 of 3451), respectively. The proportion of physicians who self-reported a clinically significant medical error within the past year by category of burnout and sleep-related impairment is illustrated in Figure 2. Had these observed associations been due to sleep-related impairment and burnout causing self-reported medical error, 37.7% of attending physicians (116 of 308) and 39.9% of trainee physicians (221 of 553) who reported making a self-reported medical error resulting in patient harm would have been able to avoid making those errors in the absence of high sleep-related impairment and high burnout.

## Discussion

These data suggest that the mean sleep-related impairment scale score in physicians was 16.9, which is modestly greater than 16, the scale score that corresponds to a T score of 50.3 (51st percentile) in combined general and clinical populations that compose PROMIS national reference data.^44,45,46^ Mean sleep-related impairment scale score in trainee physicians was 20.7. We observed a high sleep-related impairment level in physicians in surgery training programs relative to postgraduate trainees in other specialties. We also observed low sleep-related impairment in practicing surgeons relative to practicing physicians in other specialties. Together, these findings suggest that ubiquitous sleep-related impairment in surgery training programs may be related to training tradition more than to factors intrinsic to the practice of surgery and therefore may not be necessary. The results of this study suggest robust associations between sleep-related impairment, burnout, and decreased professional fulfillment and a dose-response association between sleep-related impairment and self-reported clinically significant medical error (error within the last year resulting in patient harm). The correlation between sleep-related impairment and burnout was large among attending physicians and larger among physicians in postgraduate training. The association between sleep-related impairment and self-reported clinically significant medical error persisted after controlling for burnout in a multivariate model, demonstrating that both sleep-related impairment and burnout may be independent risk factors for clinically significant error. These results are congruent with previous research indicating that sleep-related impairment—along with burnout and low professional fulfillment in physicians—is associated with increased unsolicited patient complaints.^38,47^ If observed associations were due to sleep related impairment and burnout causing error, strategies to mitigate these wellness factors could reduce medical error. 

**Figure 3.**
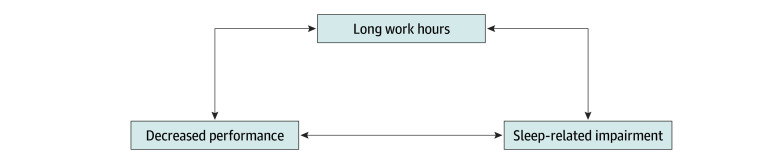
Cycle of Sleep-Related Impairment in Medicine Occupational sleep-related impairment has been associated with a reduced capacity for attention, which could contribute to the cycle illustrated in this figure, in which sleep deprivation from excessive work hours could lead to decreased efficiency, which consequently could contribute to increased work hours. Reciprocal associations between these are also possible, as indicated by the bidirectionality of the arrows in this figure.

In addition to placing patients at potential risk, high levels of sleep-related impairment place physicians at elevated personal health risk. A myriad of negative effects of inadequate sleep are well documented. Chronic inadequate sleep is associated with risk of Alzheimer disease via several mechanisms: decreased clearance of extracellular metabolites, including amyloid-beta, increased oxidative stress, and disrupted function of the blood-brain barrier.^48^ Inadequate sleep is also associated with impairments in cardiovascular health,^49^ mood,^50,51,52^ inflammatory responses, immune function,^53,54,55,56^ attention,^18,19,57^ emotion processing,^10^ and affect regulation.^9,10^ These findings suggest a credible mechanism accounting for the effect of inadequate sleep on burnout^58^: sleep deprivation leads to reduced cognitive performance coupled with impaired mood and affect regulation, thus contributing to emotional exhaustion and interpersonal disengagement. This mechanism may also add to the explanation of why burnout is an occupational syndrome^1^ for which physicians are at particular risk, given their extensive hours and shift work.^2,59,60,61,62,63,64,65,66^ Because work-related sleep impairment leads to exhaustion, a reduced capacity for attention may contribute to a vicious circle: sleep deprivation from excessive work hours may decrease efficiency, which contributes to increased work hours (Figure 3). It is also possible that conscious involvement in a medical error that resulted in patient harm contributes to the development of burnout, sleep-related impairment, or both.

Physicians may also face elevated risk of burnout because their occupation often requires high-level emotional processing, which may be difficult to accomplish during sleep deprivation. Neuroimaging evidence demonstrates hyperreactivity in the sleep-deprived amygdala,^9,11,12,67,68^ suggesting increased emotional intensity after sleep deprivation; 1 night of sleep deprivation results in 60% greater amygdala activity in response to adverse imagery.^10^ Experiencing amplified reactivity may be particularly challenging for physicians and physicians in training because of their ongoing exposure to death, trauma, and disease in medical practice. Further research on effective interventions to mitigate sleep-related impairment is warranted for both physicians and their patients. Potential directions include large-scale regulation at state and national levels, congruent with initiatives at individual, departmental, and institutional levels. Strategies may include limiting the length and frequency of extended shifts, minimizing the number of successive night shift or on-call nights, mandating rest breaks during longer shifts, receiving melatonin supplements before night shifts, using a so-called anchor sleep schedule to maintain some overlap of sleep hours when changing shifts frequently to facilitate circadian adaptation,^69^ and changing the cultural narrative around sleep in medicine.

The gap in sleep-related impairment levels between attending physicians and physicians in training suggests that the latter and the patients they care for may face particularly high risk.^70^ Inadequate sleep may exacerbate high levels of stress during postgraduate medical training. Research is needed on the costs of sleep-related impairment in academic medical centers charged with innovation, compassionate patient care, and education of new generations of physicians.

### Limitations

This study has limitations. The sample of physicians at academic-affiliated medical centers may not be sufficiently representative to render generalizable results for all physicians. Furthermore, physicians who did not respond may experience different levels of sleep-related impairment and other factors relevant to this study. Data deidentification by a third-party survey administrator removed institutional identifiers, rendering analysis of response patterns by response rate variance by institution impossible. Furthermore, assessment times of the current study included a 1-week period for sleep-related impairment and as long as 1 year for self-reported clinically significant medical error, additionally complicating interpretation of the results. In addition, the cross-sectional nature of our data did not permit cause-and-effect assessment. However, substantial extant literature reporting the effects of inadequate sleep on cognitive and clinical performance renders a strong causal relationship plausible.^9^ It is also possible that conscious involvement in a medical error that resulted in patient harm contributed to the development of burnout, sleep-related impairment, or both. Previous longitudinal research suggests these associations are reciprocal.^47,71^ The strong observed associations warrant further investigation.

## Conclusions

In this study, sleep-related impairment was prevalent in our sample of more than 11 000 attending and trainee physicians. It was linked to increased burnout and decreased professional fulfillment, demonstrating a dose-response association with self-reported clinically significant medical error. Further investigation is needed on effective interventions to reduce sleep-related impairment, with the goal of reducing harm to patients and physicians.
